# Clinical utilisation of multimodal quantitative magnetic resonance imaging in investigating muscular damage in Duchenne muscular dystrophy: a study on the association between gluteal muscle groups and motor function

**DOI:** 10.1007/s00247-023-05632-7

**Published:** 2023-03-09

**Authors:** Yu Song, Hua-yan Xu, Ke Xu, Ying-kun Guo, Lin-jun Xie, Fei Peng, Rong Xu, Hang Fu, Wei-feng Yuan, Zi-qi Zhou, Bo-chao Cheng, Chuan Fu, Hui Zhou, Xiao-tang Cai, Xue-sheng Li

**Affiliations:** 1 Department of Radiology, Key Laboratory of Obstetric and Gynecologic and Pediatric Diseases and Birth Defects of Ministry of Education, West China Second University Hospital, Sichuan University, Chengdu, 610041 China; 2 Department of Radiology, Second Affiliated Hospital of Nanchang University, Nanchang, China; 3 Department of Rehabilitation, Key Laboratory of Obstetric and Gynecologic and Pediatric Diseases and Birth Defects of Ministry of Education, West China Second University Hospital, Sichuan University, Chengdu, China

**Keywords:** Children, Duchenne muscular dystrophy, Fat fraction, Hip muscles, Magnetic resonance imaging, Motor function, Pelvic muscles, T1 mapping, T2 mapping

## Abstract

**Background:**

Duchenne muscular dystrophy (DMD) is a neuromuscular disease characterised by progressive muscular weakness and atrophy. Currently, studies on DMD muscle function mostly focus on individual muscles; little is known regarding the effect of gluteal muscle group damage on motor function.

**Objective:**

To explore potential imaging biomarkers of hip and pelvic muscle groups for measuring muscular fat replacement and inflammatory oedema in DMD with multimodal quantitative magnetic resonance imaging (MRI).

**Materials and methods:**

One hundred fifty-nine DMD boys and 32 healthy male controls were prospectively included. All subjects underwent MRI examination of the hip and pelvic muscles with T1 mapping, T2 mapping and Dixon sequences. Quantitatively measured parameters included longitudinal relaxation time (T1), transverse relaxation time (T2) and fat fraction. Investigations were all based on hip and pelvic muscle groups covering flexors, extensors, adductors and abductors. The North Star Ambulatory Assessment and stair climbing tests were used to measure motor function in DMD.

**Results:**

T1 of the extensors (*r* = 0.720, *P* < 0.01), flexors (*r* = 0.558, *P* < 0.01) and abductors (*r* = 0.697, *P* < 0.001) were positively correlated with the North Star Ambulatory Assessment score. In contrast, T2 of the adductors (*r* = -0.711, *P* < 0.01) and fat fraction of the extensors (*r* = -0.753, *P* < 0.01) were negatively correlated with the North Star Ambulatory Assessment score. Among them, T1 of the abductors (*b* = 0.013, *t* = 2.052, *P* = 0.042), T2 of the adductors (*b* = -0.234, *t* = -2.554, *P* = 0.012) and fat fraction of the extensors (*b* = -0.637, *t* =  − 4.096, *P* < 0.001) significantly affected the North Star Ambulatory Assessment score. Moreover, T1 of the abductors was highly predictive for identifying motor dysfunction in DMD, with an area under the curve of 0.925.

**Conclusion:**

Magnetic resonance biomarkers of hip and pelvic muscle groups (particularly T1 values of the abductor muscles) have the potential to be used as independent risk factors for motor dysfunction in DMD.

## Introduction

Duchenne muscular dystrophy (DMD) is an X-linked recessive genetic disease caused by mutation of the *dystrophin* gene [1, 2], with an incidence rate of approximately 1/3500 ~ 5000 among live-born boys [3, 4]. The dystrophin protein connects the muscle cytoskeleton with the extracellular matrix and prevents the muscle membrane from being damaged during muscle contraction [5, 6]. Therefore, loss of the dystrophin protein will lead to repeated degeneration of muscle fibres, chronic inflammation, progressive fibrosis and fat replacement [7, 8]. With increasing age, the muscle progressively atrophies, the functional decline worsens and the patient eventually dies of cardiopulmonary failure [9].

Although DMD is incurable [10, 11], many studies on DMD therapy have been conducted. Gene editing, exon skipping and stop codon readthrough have been used to regulate the expression of functional muscular dystrophy proteins. Additionally, efforts have been made to improve muscle function by rehabilitation training [12–14]. Previous research [15] showed that the affected muscles are more sensitive to rehabilitation treatment in the early stage, emphasising the necessity and practicability of using relevant sensitive biomarkers to characterise the muscles significantly associated with the patient’s motor function, which may guide rehabilitation therapists to carry out targeted training. Therefore, there is an urgent need for objective, highly specific and sensitive biomarkers for noninvasively characterising disease progression and treatment efficacy in DMD [16]. In our previous study of individual muscles, our team found that gluteus maximus is the most responsive to disease progression in DMD [17]. Similarly, previous studies on DMD muscle function mostly focused on individual muscles [18, 29, 31, 33], and little is known regarding the effect of hip and pelvic muscle group damage on motor function or the response to disease progression. Furthermore, few studies have compared the value of multimodal quantitative technologies, such as T1 mapping, T2 mapping or the Dixon technique, in precisely evaluating disease severity.

Therefore, the purpose of this study was to explore the utilisation of multimodal quantitative musculoskeletal MRI, to investigate the imaging biomarkers that would be most valuable for assessing pathological fat infiltration and oedema and to further identify targeted hip and pelvic muscle groups that are most associated with motor function in DMD.

## Materials and methods

### Study participants

**Fig. 1:**
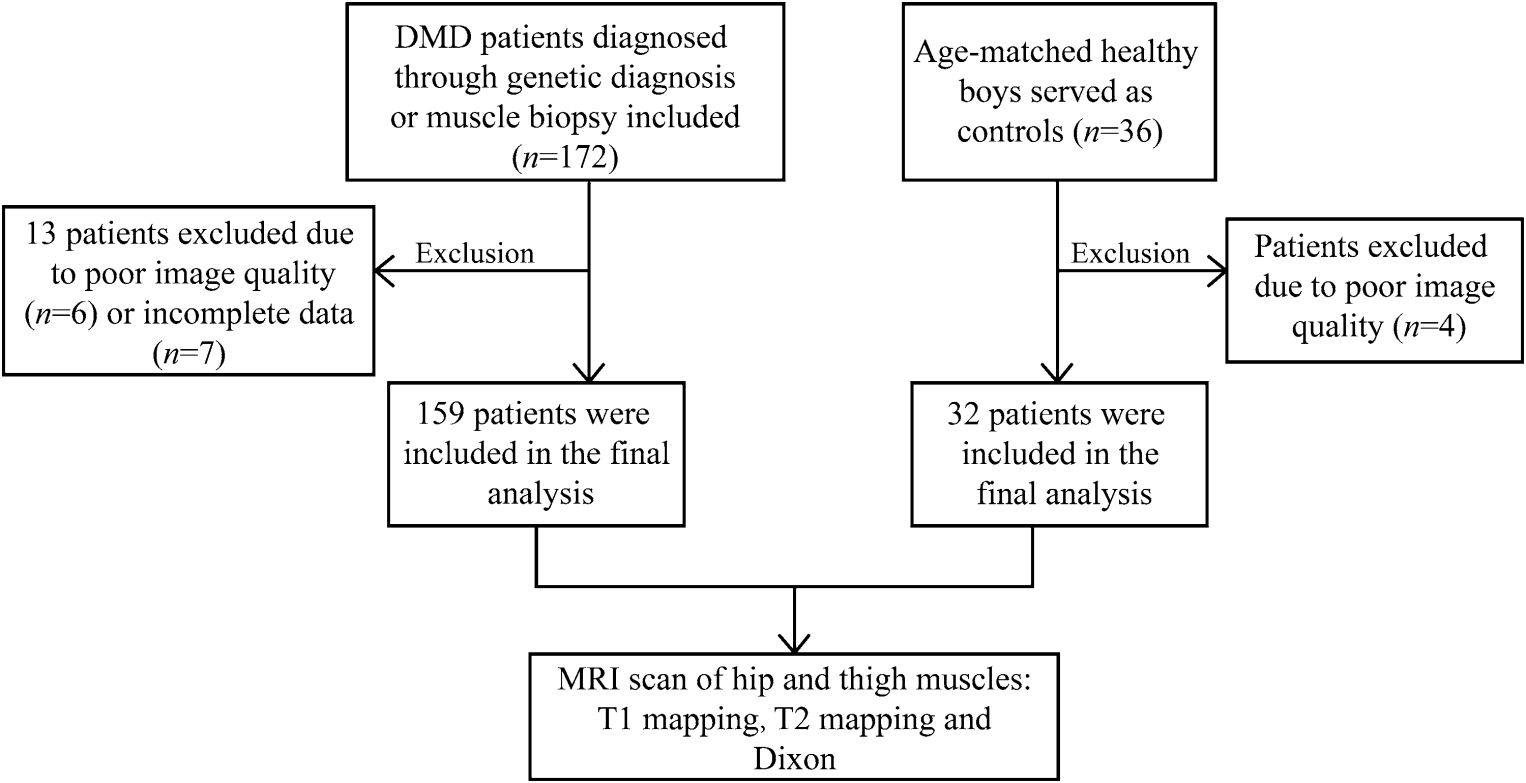
Study flow diagram. *DMD* Duchenne muscular dystrophy, *MRI *magnetic resonance imaging

From March 2020 to June 2021, we prospectively included 159 children with DMD diagnosed by genetic testing and/or skeletal muscle pathology. Thirty-two age-matched healthy males without muscle injury or other myopathy/rheumatism were enrolled as controls (Fig. 1). The exclusion criteria were an inability to cooperate during the MRI examination resulting in inadequate MRI image quality. Baseline demographic and clinical characteristics were recorded in all patients. This prospective study was reviewed and approved by the Institutional Review Board (IRB), and prior informed consent was obtained from the subjects’ guardian.

### Image acquisition

Imaging was performed with a 3-tesla (T) MR scanner (Magnetom Skyra, Siemens Healthineers, Erlangen, Germany) equipped with an 18-channel receiver coil. All patients underwent MRI scanning ranging from the iliac crest to the mid-thigh. Magnetic resonance imaging protocols included T1 mapping, T2 mapping and Dixon sequences. Sequence parameters were set as follows: T1 mapping — modified Look-Locker inversion recovery (MOLLI) sequence (echo time (TE) = 1.1 ms, repetition time (TR) = 2.7 ms, flip angle (FA) = 35°, slice thickness = 6 mm, matrix = 400 × 340, acquisition time (T_acp_ = 54 s)); T2 mapping — 3 echo times (TEs) = 0 to 55 ms (0/30/55 ms), TR = 3 ms, FA = 12°, slice thickness = 6 mm, matrix = 410 × 330 and T_acp_ = 54 s; Quantitative water/fat imaging (T2-weighted Dixon sequencing — fast spin echo (TE = 55 ms, TR = 3660 ms, FA = 150°, slice thickness = 6 mm, matrix = 360 × 360 and T_acp_ = 2 min 34 s).

### Clinical motor function assessments

All enrolled children with DMD underwent clinical motor function assessment by a paediatric neurologist using the North Star Ambulatory Assessment tool, a 17-item measure of ambulatory function with a score range of 0 to 34 (the higher the score, the better the motor function) [19]. Patients with scores below 13 may lose their walking ability within 2 years [20]. For patients with a score of 18 or more, walking ability is likely to be retained for 2 years [21]. The North Star Ambulatory Assessment raw score is converted into a more objective linear score ranging from 0 to 100 by Rasch analysis [22]. All DMD patients also underwent a stair climbing test and were divided into two subgroups based on their ability to climb the stairs: the functional stability group and the dysfunctional group. Among them, the functional ability group consisted of those patients who were able to climb stairs, either independently or with external support, while the dysfunctional ability group consisted of those unable to climb stairs.

### Imaging assessments

**Fig. 2:**
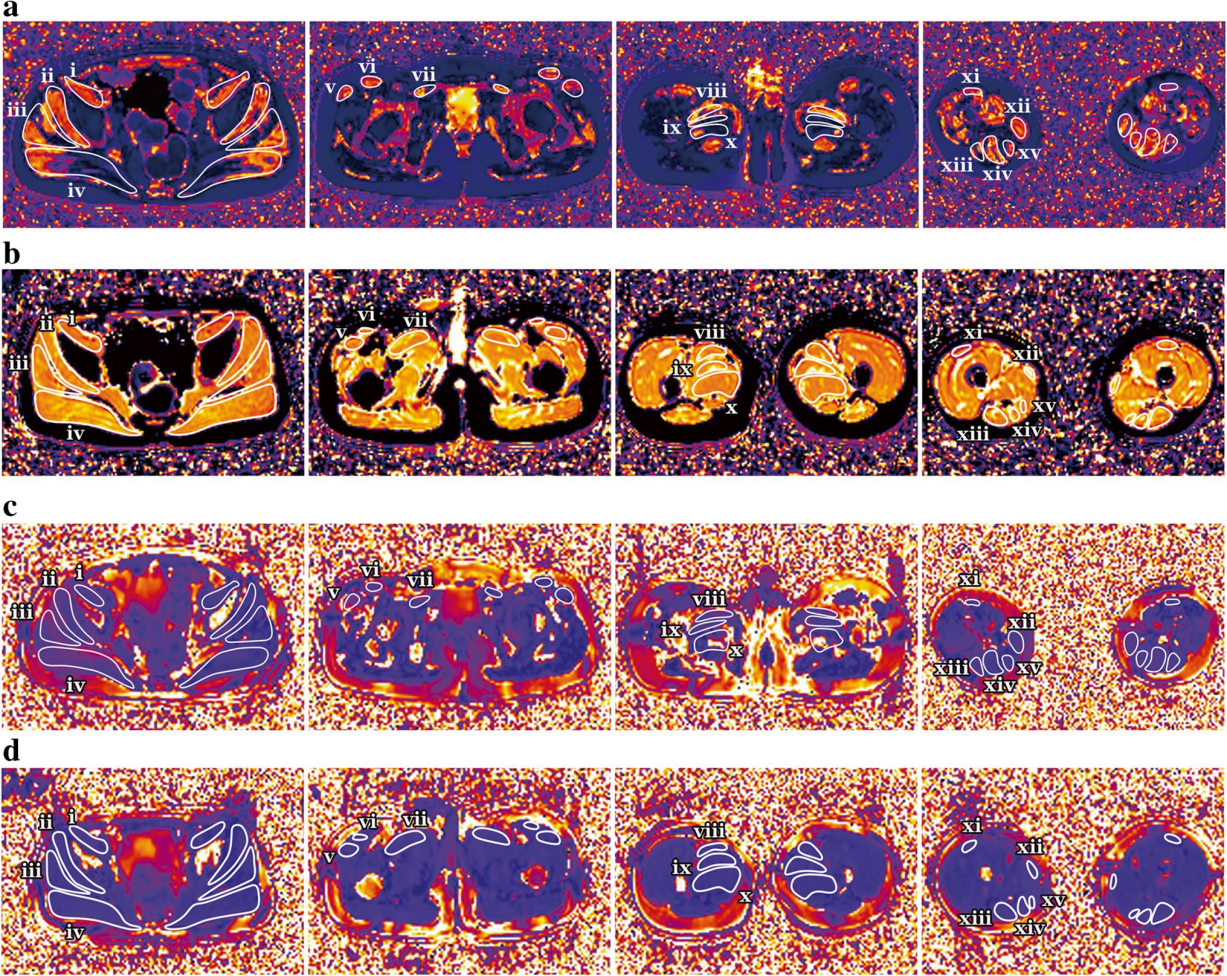

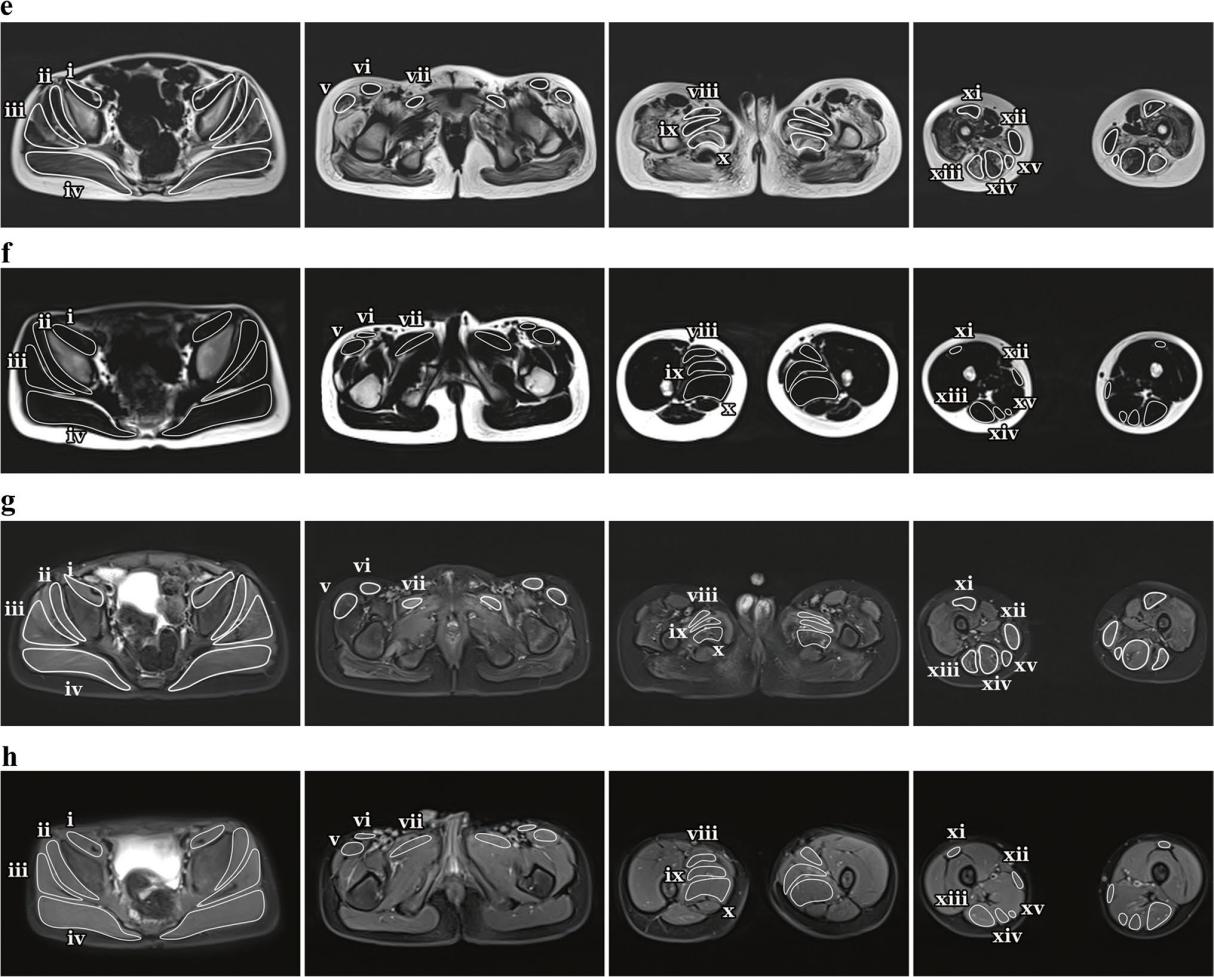
**a**–**h** Axial magnetic resonance imaging of the hip and pelvic muscles in a 10-year-old boy with Duchenne muscular dystrophy (**a**, **c**, **e**, **g**) and in an 8-year-old healthy boy (**b**, **d**,** f**,** h**). Sequences shown are T1 maps (**a**, **b**), T2 maps (**c**, **d**), Dixon (F) (**e**,** f**) and Dixon (W) (**g**, **h**). *i* iliacus, *ii* gluteus minimus, *iii* gluteus medius, *iv* gluteus maximus, *v* tensor fascia lata, *vi* sartorius, *vii* pectineus, *viii* adductor longus, *ix* adductor brevis, *x* adductor magnus, *xi* rectus femoris, *xii* gracilis, *xiii* biceps femoris, *xiv* semitendinosus, *xv* semimembranosus

Data measurement was performed by two experienced radiologists (Y.S., a radiologist with 5 years of experience and F.P., a radiologist with 8 years of experience) independently using a Siemens MR Post-Processing workstation (Syngo.Via, Erlangen, Germany), and one radiologist (Y.S.) repeated the measurements 2 weeks later. Investigations were all based on muscle groups covering the flexors (iliacus, rectus femoris and sartorius), extensors (gluteus maximus, biceps femoris, semitendinosus and semimembranosus), adductors (adductor magnus, adductor longus, adductor brevis pectineus and gracilis) and abductors (gluteus medius, gluteus minimus and tensor fascia lata). Slices that contained the largest area of visible muscle with good differentiation of diverse muscle groups were chosen for placing the region of interest (ROI), including four major cross-sectional levels: (a) level near the sciatic foramen; (b) level near the greater trochanter-ischial tuberosity; (c) level near the proximal part of the femoral diaphysis; and (d) level near the middle of the femoral diaphysis (Fig. 2). The T1 and T2 maps were colour coded pixel by pixel with colours corresponding to a range of T1 and T2 values. Placement of the ROI of each muscle on the T1 or T2 map allowed automatic calculation of the mean T1 or T2 value. Fat fraction values were calculated as signal intensity (SI) fat/(SI _fat_ + SI _water_) × 100% from the reconstructed fat and water images [23]. The size of the ROI was determined by using the individual muscle size on the axial images. The average T1, T2 and fat fraction values were calculated using the ROIs outlined on three consecutive slices of each muscle. The T1, T2 and fat fraction values of the hip and pelvic muscle groups were obtained as follows: Firstly, the muscles that make up flexor, extensor, adductor and abductor are outlined. Secondly, the average of these quantitative MRI indicators of each muscle is the corresponding quantitative MRI indicator of each hip and pelvic muscle group.

### Statistical analysis

Statistical analysis was performed using Statistical Product and Service Solutions software (SPSS, version 26.0, IBM Crop, Armonk, NY, USA) and MedCalc software (MedCalc Software Ltd, Ostend, Belgium). Data that followed the normal distribution were expressed as the mean ± standard deviation; those that did not follow the normal distribution were expressed as the median (interquartile range). Comparison of continuous variables was performed using either Student’s *t* test or the Mann–Whitney *U* test as appropriate. Kruskal Wallis rank sum test was used to compare the MR biomarkers in different DMD age subgroups. Spearman correlation coefficients were used to evaluate the correlation between quantitative MRI indicators of muscle groups and the North Star Ambulatory Assessment score. Linear regression analysis was performed to determine the influence of the quantitative MRI indicators of muscle groups on the North Star Ambulatory Assessment score. Receiver operator characteristic (ROC) curve analysis was performed to investigate the diagnostic value of T1 of the abductors, T2 of the adductors and fat fraction of the extensors, and the area under the curve (AUC) was calculated. Consequently, Youden’s index was used to calculate the optimal cut-off value for DMD. Interclass correlation coefficient (ICC) was used to determine the intra- and inter-rater reliability of the MRI indicators by ROI measurement.

## Results

### Participant population

Details of screening, exclusion and eligibility for analysis of this study population are depicted in the study flow chart (Fig. 1). A total of 172 patients with DMD were prospectively enrolled. After excluding 13 patients with poor image quality (*n* = 6) or incomplete data (*n* = 7), 159 patients with DMD were finally included (functional ability group = 125; dysfunctional ability group = 34). All patients with DMD were divided into three age subgroups: 5–8-, 9–12- and 13–15-year-old children, respectively, of 72, 67 and 20 subjects each. A further 32 healthy boys were included as controls. The baseline characteristics of the study population are summarised in Table 1.

**Table 1:** Baseline characteristics of the study population

Clinical characteristics	DMD (*n* = 159)	Controls (*n* = 32)	*P-*value
Age, years (y)	9.08 (2.67)	9.46 (2.23)	0.744
Age, minimum/maximum, y	4.92/15.00	6.00/16.00	*NA*
Age 25 ~ 75%, y	7.58 ~ 9.56	8.17 ~ 10.40	*NA*
Male, %	100	100	*NA*
Height, cm	125.00 (16.50)	135.19 ± 14.77	0.004
Weight, kg	27.00 (11.50)	29.25 (13.00)	0.146
BMI, kg/m^2^	17.53 (3.84)	15.57 (3.56)	0.04
Corticosteroid use, *n*^a^	128/31	0/32	*NA*
Wheelchair use, *n*^a^	16/143	0/32	*NA*
NSAA score	51.75 ± 27.33	*NA*	*NA*
Functional stability group/dysfunctional group	125/34	32/0	*NA*

*P*-values reflect comparisons between DMD and controls

Values are presented as the mean ± standard deviation or median (interquartile range)

*BMI* body mass index, *DMD* Duchenne muscular dystrophy, *NA* not available, *NSAA* North Star Ambulatory Assessment

^a^For corticosteroid use, values represent the following statuses: on/off

^b^For wheelchair use, values represent the following statuses: on/off

### Comparison of magnetic resonance biomarkers

**Fig. 3:**
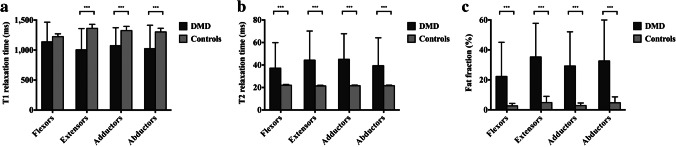
Comparison of magnetic resonance biomarkers of hip and pelvic muscle groups between Duchenne muscular dystrophy (DMD) patients and controls. **a** T1 of the extensors, adductors and abductors in DMD were significantly lower than in controls. **b** T2 of the flexors, extensors, adductors and abductors in DMD were significantly higher than in controls. **c** Fat fraction of the flexors, extensors, adductors and abductors in DMD were significantly higher than in controls. Significant differences are marked as ***P < 0.001

**Fig. 4:**
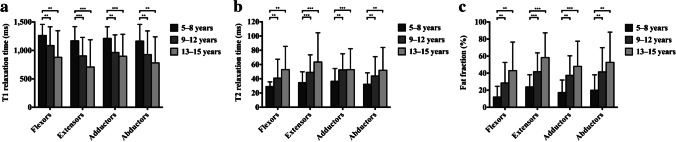
Comparison of magnetic resonance biomarkers of hip and pelvic muscle groups between different Duchenne muscular dystrophy age subgroups. **a** The T1 values in the 5–8-year age subgroup was significantly higher than that in the 9–12- and 13–15-year age subgroups. **b** The T2 values in the 5–8-year age subgroup was significantly lower than those in the 9–12- and 13–15-year age subgroups. **c** The fat fraction values in the 5–8-year age subgroup was significantly lower than those in the 9–12- and 13–15-year age subgroups. Significant differences are marked as ***P < 0.001, **P < 0.01, *P < 0.05

The comparison of the quantitative MRI indicators (T1, T2 and fat fraction) of the flexors, extensors, adductors and abductors of DMD patients and controls is shown in Fig. 3. Compared with that of the controls, the T1 of the extensors, adductors and abductors of DMD was significantly lower (*P* <0.001). The T2 and fat fraction of the flexors, extensors, adductors and abductors were significantly higher than those of the controls (*P* < 0.001) (Table 2). Figure 4 illustrates the comparison of T1, T2 and fat fraction values of hip and pelvic muscle groups at different DMD age subgroups. We found that as the age group increased, T1 values of muscle groups showed a gradually decreasing trend, while T2 and fat fraction values showed a gradually increasing trend. The changes in MRI indicators differ between DMD age subgroups. The T1 values in the 5–8-year-old age subgroup was significantly higher than that in the 9–12- and 13–15-year-old age subgroups (*P*<0.05), but there was no significant difference between the 9–12- and 13–15-year-old age subgroups (*P* >0. 05). The T2 and fat fraction values in the 5–8-year-old age subgroup were significantly lower than those in the 9–12- and 13–15-year-old age subgroups (*P*<0.05), but there was no significant difference between the 9–12- and 13–15-year-old age subgroups (*P* >0.05).

**Table 2:** T1, T2, and fat fraction of hip and pelvic muscle groups in Duchenne muscular dystrophy (DMD) patients and controls

	Muscle groups	DMD	Control group	Z	*P-*value
T1 (ms)	Flexors	1234.755 (287.2)	1223.136 ± 47.744	−0.289	0.773
	Extensors	1074.84 (442.89)	1361.484 ± 67.766	−6.628	<0.001
	Adductors	1146.195 (455.66)	1300.917(72.96)	−5.132	<0.001
	Abductors	1175.385 (640.79)	1285.088(82.14)	−3.771	<0.001
T2 (ms)	Flexors	29.21 (11.24)	21.973 ± 0.773	−8.043	<0.001
	Extensors	32.48 (29.36)	21.397 ± 0.594	−8.125	<0.001
	Adductors	35.3 (30.09)	21.561 ± 0.583	−8.504	<0.001
	Abductors	29.65 (22.27)	21.595 ± 0.488	−7.605	<0.001
Fat fraction (%)	Flexors	16.06% (24.31%)	2.425%(1.32%)	−7.101	<0.001
	Extensors	30.49% (30.61%)	2.925%(2.97%)	−8.054	<0.001
	Adductors	22.95% (34.80%)	2.515%(1.69%)	−7.909	<0.001
	Abductors	22.88% (41.62%)	3.76%(3.54%)	−7.192	<0.001

*P*-values reflect comparisons between DMD and controls

Values are presented as the mean ± standard deviation or median (interquartile range)

### Correlation between the North Star Ambulatory Assessment score and magnetic resonance biomarkers

**Fig. 5:**
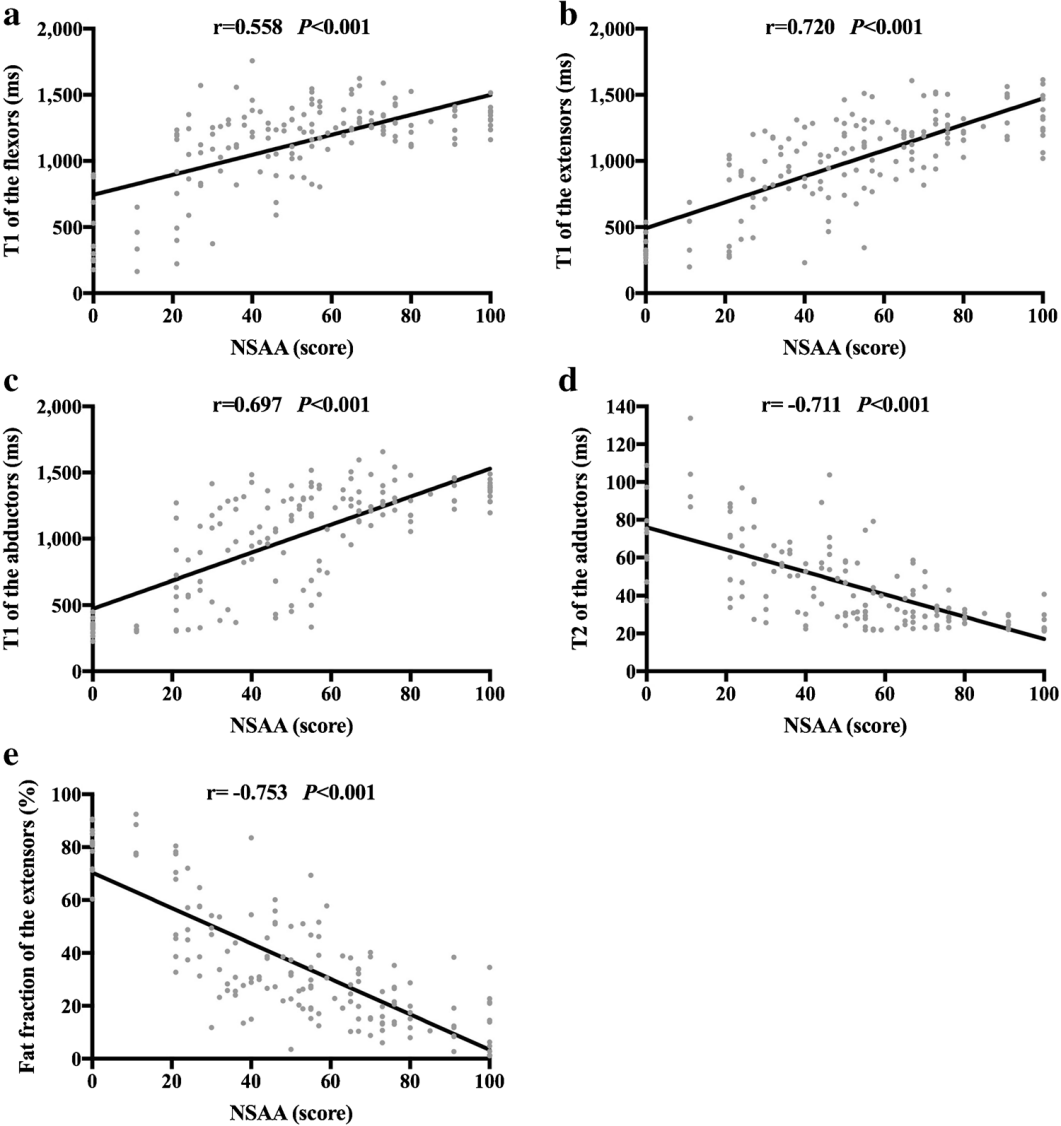
Scatterplots demonstrating the association between the magnetic resonance biomarkers and the North Star Ambulatory Assessment (NSAA) score. **a** T1 of the flexors showed a moderate positive correlation with the NSAA score. **b** T1 of the extensors showed a strong positive correlation with the NSAA score. **c** T1 of the abductors showed a moderate positive correlation with the NSAA score. **d** T2 of the adductors showed a strong negative correlation with the NSAA score. **e** Fat fraction of the extensors showed a strong negative correlation with the NSAA score

There was a strong positive correlation between the T1 of the extensors and the North Star Ambulatory Assessment score (*r* = 0.720, *P* < 0.001), while there was a strong negative correlation between the T2 of the adductors and fat fraction of the extensors and the North Star Ambulatory Assessment score (*r* = -0.711, *P* < 0.001; *r* = -0.753, *P* < 0.001). Moderate positive correlations were detected between the T1 of the flexors and abductors and the North Star Ambulatory Assessment score (*r* = 0.558, *P* < 0.001; *r* = 0.697, *P* < 0.001) (Fig. 5).

### Linear regression analysis of North Star Ambulatory Assessment score and magnetic resonance biomarkers

Table 3 shows the multivariable models. The T1 of the flexors, extensors and abductors, T2 of the adductors and fat fraction of the extensors were included to construct a linear regression equation (model 1). Variables in model 1 with *P* < 0.05 are included in model 2: T1 of the abductors, T2 of the adductors and fat fraction of the extensors were included to construct a linear regression equation. The results showed that the T1 of the abductors significantly positively affected the North Star Ambulatory Assessment score (*b* = 0.012, *P* = 0.037), while the T2 of the adductors (*b* = -0.223, *P* = 0.013) and fat fraction of the extensors (*b* = -0.590, *P* < 0.001) significantly negatively affected the North Star Ambulatory Assessment score. The results also showed that when the T1 of the abductors increased by 1 ms, the North Star Ambulatory Assessment score increased by 0.012 points. When the T2 of the adductors increased by 1 ms, the North Star Ambulatory Assessment score decreased by 0.223 points; when the fat fraction of the extensors increased by 1%, the North Star Ambulatory Assessment score decreased by 0.590 points. Our results indicate that T1 of the abductors, T2 of the adductors and fat fraction of the extensors are independent factors influencing the North Star Ambulatory Assessment score of patients with DMD.

**Table 3:** Independent influencing factors of the NSAA score in DMD

	Model 1				Model 2			
	*B*	Beta	*t*	*P* value	*B*	Beta	*t*	*P*-value
T1 of the flexors	−0.007	−0.081	−0.889	0.375				
T1 of the extensors	0.003	0.046	0.369	0.713				
T1 of the abductors	0.014	0.214	2.221	0.028	0.012	0.182	2.102	0.037
T2 of the adductors	−0.235	−0.202	−2.557	0.012	−0.223	−0.192	−2.506	0.013
Fat fraction of the extensors	−0.574	−0.488	−3.923	<0.001	−0.590	−0.501	−6.187	<0.001

Model 1: T1 of the flexors, extensors, and abductors, T2 of the adductors, and fat fraction of the extensors were included to construct a linear regression equation. Variables in Model 1 with *P* < 0.05 are included in Model 2: T1 of the abductors and T2 of the adductors and fat fraction of the extensors were included to construct a linear regression equation

*B* unstandardised coefficient, *Beta* standardised coefficient, *DMD* Duchenne muscular dystrophy, *NSAA* North Star Ambulatory Assessment, *VIF* variance inflation factor

### Diagnostic value of magnetic resonance biomarkers for predicting Duchenne muscular dystrophy severity

**Fig. 6:**
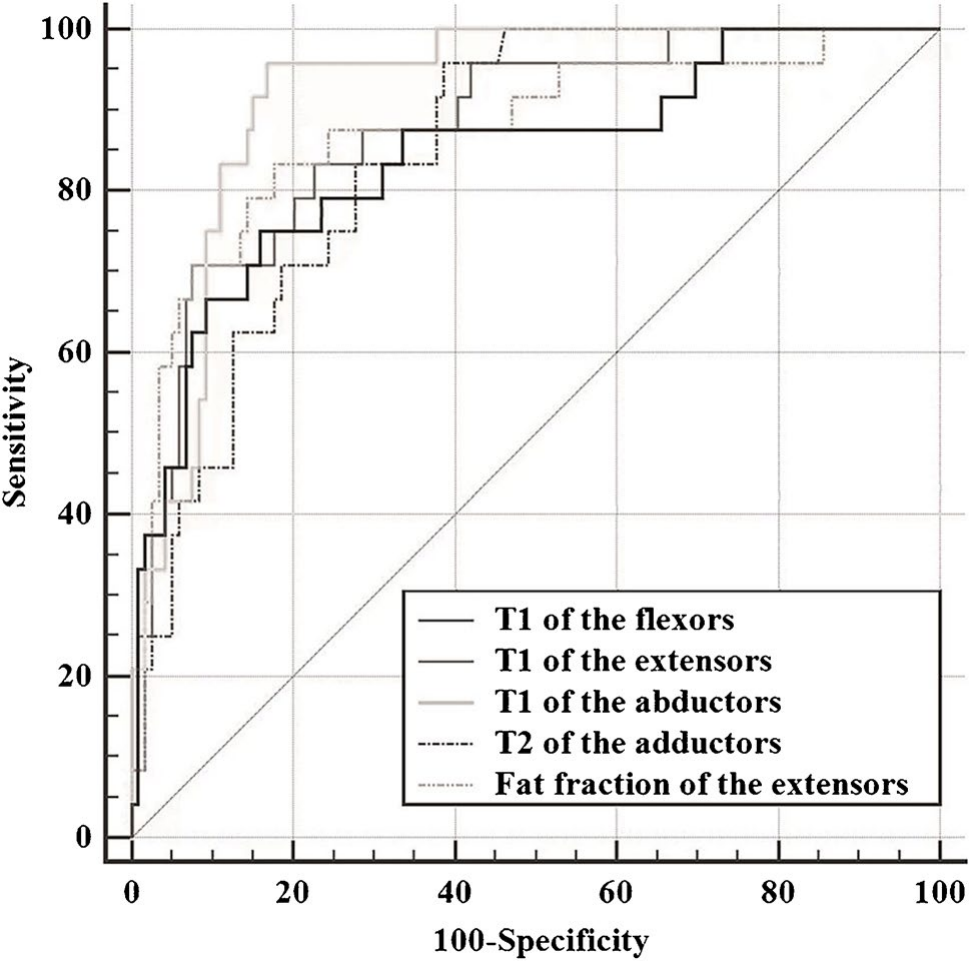
Receiver operating characteristic curves for the T1 of the flexors, T1 of the extensors, T1 of the abductors, T2 of the adductors and fat fraction of the extensors in distinguishing Duchenne muscular dystrophy patients into functional and dysfunctional ability groups

Receiver operating curve analysis (Fig. 6) was performed to investigate the diagnostic value of T1 of the flexors, extensors and abductors, T2 of the adductors and fat fraction of the extensors in the DMD subgroups (functional ability group/dysfunctional group). The ROC analysis (Table 4) showed that T1 of the abductors was highly predictive for identifying the severity of motor function in DMD patients, T1 of the flexors, T1 of the extensors, T2 of the adductors and fat fraction of the extensors were moderately predictive for identifying the severity of motor function in DMD patients, with AUC of 0.925, 0.834, 0.888, 0.857 and 0.885, respectively. Youden’s index was 0.798, 0.568, 0.642, 0.578 and 0.653, respectively. Concerning T1 of the abductors, a cut-off value of  <895.4 ms can distinguish patients with motor dysfunction from those with stable motor function, with a sensitivity of 96.3% and specificity of 83.5%. Concerning T1 of the flexors, a cut-off value of  <1075.5 ms can distinguish patients with motor dysfunction from those with stable motor function, with a sensitivity of 72.0% and specificity of 84.8%. Concerning T1 of the extensors, a cut-off value of  <732.4 ms can distinguish patients with motor dysfunction from those with stable motor function, with a sensitivity of 71.4% and specificity of 92.8%. Concerning T2 of the adductors, a cut-off value of  >35.6 ms can distinguish patients with motor dysfunction from those with stable motor function, with a sensitivity of 96.0% and specificity of 61.8%. Concerning fat fraction of the extensors, a cut-off value of  >43.8% can distinguish patients with motor dysfunction from those with stable motor function, with a sensitivity of 82.1% and specificity of 83.2%.

**Table 4:** Receiver operating  characteristic curve analysis of magnetic resonance biomarkers for predicting Duchenne muscular disease severity

	Cut-off	Area under the curve (95%CI)	Sensitivity	Specificity	Youden index
T1 of the flexors	1075.475	0.834 (0.740 ~ 0.928)	72.00%	84.80%	0.568
T1 of the extensors	732.390	0.888 (0.823 ~ 0.952)	71.40%	92.80%	0.642
T1 of the abductors	895.471 ms	0.925 (0.870 ~ 0.962)	96.30%	83.47%	0.798
T2 of the adductors	35.630 ms	0.857 (0.791 ~ 0.909)	96.00%	61.79%	0.578
Fat fraction of the extensors	43.786%	0.885 (0.824 ~ 0.931)	82.14%	83.20%	0.653

A comparison of the ROC curves revealed that the AUC of T1 of the abductors was significantly higher than that of T2 of the adductors (95% CI: 0.016 ~ 0.129, *Z* = 2.516, *P* = 0.012) in identifying motor dysfunction in DMD patients, while there was no significant difference between the other groups. That is, T1 of the abductors has higher diagnostic value for predicting the severity of DMD.

### Intra- and inter-observer reliability of magnetic resonance biomarkers

The ICC analysis showed excellent intra- and inter-rater reliability of MRI indicators by ROI measurement. The range of ICC for intra- and inter-rater reliability for T1 values was 0.959 ~ 0.984 and 0.941 ~ 0.984, respectively. The range of ICC-intra- and ICC-inter-rater reliability for T2 values was 0.904 ~ 0.956 and 0.920 ~ 0.957, respectively. The range of ICC for intra- and inter-rater reliability for fat fraction was 0.982 ~ 0.997 and 0.986 ~ 0.994, respectively.

## Discussion

In the present study, we demonstrate that (a) T1 of the extensors and abductors (positively), T2 of the adductors (negatively) and fat fraction of the extensors (negatively) were strongly correlated with the North Star Ambulatory Assessment score; (b) decreased T1 of the abductors, increased T2 of the adductors and fat fraction of the extensors are independent risk factors for motor dysfunction in DMD; and (c) T1 of the abductors has higher diagnostic value for the DMD disease severity.

Multimodal quantitative MRI can reflect the different physiological characteristics of muscle. Fibrosis and inflammation lead to changes in the molecular composition of tissues, resulting in increased T1 [24, 25], while fatty infiltration decreases T1 [26]. T2 can objectively quantify muscle oedema and fat infiltration. Water molecule motion restriction leads to signal decay, resulting in increased T2 [27]. Fat fraction reflects muscle fat infiltration, with a higher value indicating more severe fat infiltration [28, 29].

Magnetic resonance biomarkers deteriorate over time in DMD, suggesting that focusing on this in the early stage of the disease may be important for targeted treatment and slowing disease progression. Consistent with previous studies, we found that muscle T2 and fat fraction values of DMD are higher than in healthy individuals [30, 31]. Moreover, as the disease progresses, so do MRI indicators [15, 32]. Given these variations, it is important to determine which MRI indicator is the most sensitive to changes in the hip and pelvic muscles. In our previous study of individual muscles [33], our team found that the muscle T1 values positively correlated with the North Star Ambulatory Assessment score and decreased as the grade of fat infiltration increased. Based on this research, we divided the hip and pelvic muscles into four groups for analysis. In addition, two other MRI indicators (T2 and fat fraction) were introduced. There was a significant correlation between MRI indicators (T1 of the extensors and abductors, T2 of the adductors and fat fraction of the extensors) and the North Star Ambulatory Assessment score, suggesting that multimodal quantitative MRI can detect DMD musculoskeletal lesions well, which is of great significance for accurately quantifying the disease severity. The results indicate that the decreased T1 of the extensors and abductors means a decrease in motor function. Similar findings were reported by Liu et al. [26], who discovered that the muscle T1 values in GNE myopathy (a disease caused by pathogenic variations of the *GNE* gene) decreased with disease progression. Conversely, muscle oedema or fat infiltration lead to an increase in T2 or fat fraction value, indicating a decrease in motor function, especially in adductors and extensors. A systematic review [34] reported the strongest correlation between MR biomarkers (T2 and fat fraction) of lower limb muscles and motor function. However, our study provides the first assessment of hip and pelvic muscle groups in relation to functional ability and shows that quantitative MRI indicators are associated with motor function, which can explain the involvement of muscle from the perspective of dominant activity and predict the change in its corresponding motor function. These results indicate the importance of not only focusing on specific hip and pelvic muscle groups but also considering quantitative MRI indicators when assessing severity of disease in DMD.

The second important finding of the present study was that T1 of the abductors, T2 of the adductors and fat fraction of the extensors were independent risk factors for motor dysfunction in DMD patients. Moreover, T1 of the abductors was highly predictive for identifying DMD severity. A T1 of the abductors less than 895.5 ms may predict loss of the ability to climb stairs or other dysfunction. T1 of the flexors and extensors, T2 of the adductors and fat fraction of the extensors are also worth considering. Based on these findings and given the heterogeneity of muscle groups, it is reasonable to deduce that the MR biomarkers corresponding to these muscle groups may be different when predicting motor function due to different involved patterns. Clinicians should note when any one of these biomarkers approaches or exceeds its cut-off value, particularly T1 of the abductors. Muscle MR biomarkers are highly sensitive and specific in the risk stratification of DMD, remaining valuable in identifying pathological oedema or fat infiltration in the clinic where there is no clinically apparent muscle weakness. Compared with the individual muscles reported in previous studies [30, 35], the MR biomarkers of the studied hip and pelvic muscle groups can more intuitively reflect which motor function is most obviously affected, which may provide guidance for clinicians to conduct early rehabilitation training and targeted therapy for DMD patients. Furthermore, the sensitive MR biomarkers discovered in our study can also be used to detect different characteristics of muscle pathology across the whole range of disease from pre-symptom to end-symptom, which is difficult using existing clinical tests – and the latter do not evaluate specific hip and pelvic muscle groups.

In brief, our results show that MR biomarkers of hip and pelvic muscle groups (particularly T1 of the abductors) have the potential to be used as independent risk factors for clinical motor dysfunction. Although there are currently no recognised reference values for quantitative MRI indicators for predicting DMD severity, multimodal quantitative MRI has great potential for evaluating DMD muscular dysfunction in the future.

There are some limitations in our study. First, this study does not include follow-up data and the progression of MR biomarkers over time is worthy of further investigations in future study. Second, our study selected the most representative four levels of muscle for data measurement rather the whole muscle. Third, there has been no validation of our cut-off values in other institutions. Our future research will include multicentre studies.

## Conclusion

In summary, our study reveals that MR biomarkers of hip and pelvic muscle groups have the potential to serve as independent risk factors for motor dysfunction in DMD. Specifically, T1 of the abductors has higher diagnostic value for DMD muscle group damage, suggesting that T1 may be a valuable biomarker and the abductors the most appropriate muscle group for evaluating DMD severity. Conjoint utilisation of multimodal MRI for measuring fat replacement and oedema of hip and pelvic muscle groups may help in the risk stratification of boys with DMD and further provide guidance for clinical treatment.

## Data Availability

All datasets during this study are available from the corresponding author on reasonable request.
